# Convalescent Plasma Therapy in Immunocompromised Patients Infected With the BA.1 or BA.2 Omicron SARS‐CoV‐2

**DOI:** 10.1111/irv.13272

**Published:** 2024-03-19

**Authors:** Quentin Richier, Benjamin De Valence, Dorothée Chopin, Emmanuelle Gras, Laura I. Levi, Yasmine Abi Aad, Jérôme Pacanowski, Jean‐Luc Meynard, Léo Plaçais, Dorothée Fey, Priscille Couture, Guillaume Martin‐Blondel, Vincent Pestre, Juliette Woessner, Sophie Ancellin, Pierre Weyrich, Benjamin Carpentier, Salim Idri, Pierre Tiberghien, Laure Surgers, Thomas Hueso, Karine Lacombe

**Affiliations:** ^1^ Service de Maladies Infectieuses Hôpital Saint‐Antoine, AP‐HP Paris France; ^2^ Sorbonne Université, IPLESP, Inserm UMR‐S1136 Paris France; ^3^ Département de Médecine Interne et Immunologie clinique Hôpital Bicêtre Le Kremlin‐Bicêtre France; ^4^ Service de Médecine Interne Hôpital Foch Suresnes France; ^5^ Service des Maladies Infectieuses et Tropicales Centre Hospitalier Universitaire de Toulouse Toulouse France; ^6^ INSERM UMR1043, Centre de Physiopathologie de Toulouse‐Purpan Toulouse France; ^7^ Faculté de Médecine Université de Toulouse Paul Sabatier Toulouse France; ^8^ Service de médecine interne et maladies infectieuses Centre hospitalier Avignon Avignon France; ^9^ Centre hospitalier d'Auch France; ^10^ Unité d'Infectiologie, Groupement des Hôpitaux Catholiques de Lille, F‐59160 Lille France; ^11^ Etablissement Français du Sang Créteil France; ^12^ Etablissement Français du Sang La Plaine Saint‐Denis France; ^13^ UMR 1098 RIGHT Inserm Université de Franche‐Comté, Etablissement Français du Sang Besançon France; ^14^ Service d'hématologie clinique, Hôpital Avicenne, APHP, Sorbonne Université Paris‐Nord Bobigny France

**Keywords:** convalescent plasma, COVID‐19, immunocompromised, Omicron variant, SARS‐CoV‐2

## Abstract

The emergence of SARS‐CoV‐2 Omicron variant has led to a complete reconfiguration of the therapeutic landscape, with all monoclonal antibodies having lost any neutralization activity. We report here a case series of 75 immunocompromised patients infected by the Omicron variant who benefited from COVID‐19 convalescent plasma (CCP). At Day 28, the overall survival was 76% (95% CI 67–86) with no significant difference in the clinical outcome between patients with hematological malignancies, solid organ transplantation or autoimmune diseases. No safety concern was reported during the course of the study. These results showed that CCP is well tolerated and represents a treatment option for immunocompromised patients who remain highly impacted by the COVID19 epidemic.

## 1 Introduction

Immunocompromised patients are at high risk of severe COVID‐19. Since they might not elicit an adequate immune response after vaccination, passive immunization using ex vivo produced neutralizing antibodies is one of the key therapeutic options in such population [1]. Monoclonal anti‐spike antibodies have shown to lead to a great risk reduction of hospitalization or death in immunocompetent patients [2, 3]. However, emerging Omicron SARS‐CoV‐2 variants appeared to be partially or completely resistant to available monoclonal antibodies [4]. Early treatment with COVID‐19 convalescent plasma (CCP) in unvaccinated immunocompetent patients has been shown to be associated with a lower risk of hospitalization [5]. Therefore, even if monoclonal antibodies seem associated with a greater risk reduction of disease progression than CCP, the polyclonal characteristics of CCP might be of particular interest in the context of emergence of new variants. Early in the pandemic, high‐titer CCP has shown some efficacy in B‐cell‐depleted patients [6, 7], but little is known on the efficacy of CCP in immunosuppressed patients infected by Omicron variant. Here, we report a case series of 75 immunocompromised patients infected by the BA.1 or BA.2 Omicron SARS‐CoV‐2 subvariants and treated with high‐titer Omicron CCP.

## 2 Methods

We retrospectively analyzed the data from a nationwide, observational, and multicentric study based on the French CCP Early Access Program. Between December 29, 2021, and March 16, 2022, 32 centers located in France requested the use of CCP during the SARS‐CoV‐2 Omicron variant wave. Due to underlying disease or treatment administered, patients with hematological malignancy (HM), solid organ transplanted (SOT) recipients, or those treated for autoimmune disease (AID) were considered immunosuppressed and eligible for CCP early access program. Infection with a BA.1 or BA.2 SARS‐CoV‐2 subvariants was documented on nasopharyngeal swab. We considered a threshold of positivity for anti‐spike antibodies of >264 BAU/mL, as the ability of vaccines to prevent symptomatic forms of COVID‐19 [8]. Every patient was informed of the study protocol and none refused to participate. Data were anonymized according to the French law, and ethical clearance was obtained from the French Infectious Diseases Society (CER‐MIT 2022‐0702). We used the World Health Organization (WHO) clinical progression scale for the COVID‐19 severity evaluation of the patient. Scale 5 involves hospitalized patient with oxygen by mask or nasal prongs only, and Scale 6 hospitalized patient with high flow oxygen or noninvasive ventilation [9].

We administered two consecutive transfusions of two ABO‐compatible high‐titer convalescent plasma units (200–220 mL each) at Days 0 and 1. Transfused CCP were initially provided by pre‐Omicron convalescent vaccinated donors with very high anti‐spike IgG ratio (>9, ELISA Euroimmun) to ensure anti‐Omicron seroneutralization ability [10] and after mid‐January 2022 by Omicron convalescent vaccinated donors with high‐anti spike IgG ratio (>6, ELISA Euroimmun). The primary outcome was the overall survival (OS) at Day 28 after plasma infusion (28‐day OS).

## 3 Results

Among 249 requests for CCP during the study period, 225/249 (78%) were found eligible, and 81/225 (36%) had an available follow‐up until Day 28 at time of study analysis. Six patients were subsequently excluded from the analysis: Two received sotrovimab, one was transferred in intensive care unit, two improved spontaneously, and one died before transfusion. Table 1 reports the baseline description of the 75 patients included. Most patients had received B‐cell depletion therapy such as rituximab (*n* = 51/75, 68%). Four patients who presented with two simultaneous underlying immunodeficiencies were excluded of the subgroup analysis done according to the underlying immunodeficiency. Among patient with only one underlying immunodeficiency (*n* = 71), the most frequent underlying immunodeficiency was HM (*n* = 50/71, 71%). The remaining patients were SOT recipients (*n* = 11/71, 15%) or had AID (*n* = 10/71, 14%). Of note, 91% (*n* = 68/75) of the cohort was vaccinated with at least two doses. An anti‐SARS‐CoV‐2 spike protein antibodies >264 BAU/mL was reported in eight patients (11%). Most of the patients (67/75, 89%) received CCP after mid‐January 2022. However, at that time, patients may have received units from pre‐Omicron as well as omicron era because the rule for CCP transfusion was to mix units from different donors. After the end of January, all CCP came from omicron donors.

**TABLE 1:** Baseline characteristics of the 75 immunocompromised patients infected with the BA.1 or BA.2 Omicron subvariants of SARS‐CoV‐2 and treated with CCP.

Patient baseline characteristics (*n* = 75)	
Age, years, median [IQR]	65 [59–73]
Female/male, *n*	28/47
Days from symptoms onset to CCP, days, median [IQR]	17 [10–24,5]
Initial CT level, median [IQR]	22.9 [17.5–26]
Anti‐CD20 mAb pre‐exposure (*n* = 73, unknown = 2), *n* (%)	
Yes	51 (70)
No	22 (30)
Pre exposure to monoclonal antibodies (*n* = 75), *n* (%)	
Yes	7 (9)
No	68 (91)
Two‐dose vaccination (*n* = 75), *n* (%)	
Yes	68 (91)
No	7 (9)
Initial anti‐S antibodies (*n* = 70, unknown = 5), *n* (%)	
>264 BAU/mL	8 (11)
<264 BAU/mL	62 (89)
Underlying diseases (*n* = 71, excluded patients with two simultaneous underlying immunodeficiency *n* = 4), *n* (%)	
Hematological malignancy	50 (71)
Solid organ transplant recipient	11 (15)
Autoimmune disease	10 (14)
Positive RNAemia (*n* = 18, unknown = 57), *n* (%)	
Yes	13 (72)
No	5 (28)
WHO scale (*n* = 72, unknown = 3), *n* (%)	
Scale 5	49 (68)
Scale 6	23 (32)

*Note:* WHO clinical progression scale for the COVID‐19 severity: Scale 5: *hospitalized, oxygen by mask or nasal prongs*, WHO 6: *hospitalized, oxygen by noninvasive ventilation or high flow* [9].

Abbreviations: BAU, binding antibody unit; CCP, COVD‐19 convalescent plasma; mAb, monoclonal antibody.

**FIGURE 1:**
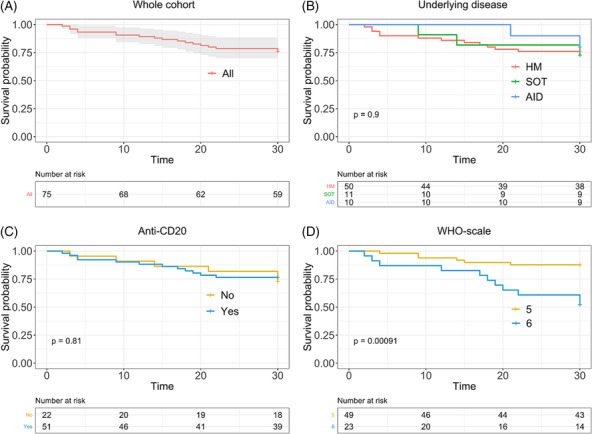
Overall survival of whole cohort (A), according to underlying disease (B), according to previous administration of anti‐CD20 monoclonal antibody (C), and according to COVID‐19 severity at the time of CCP infusion (D). AID, autoimmune disease; HM, hematological malignancy; SOT, solid organ transplant recipient. WHO scale 5: *hospitalized, oxygen by mask or nasal prongs*; WHO scale 6: *hospitalized, oxygen by noninvasive ventilation or high flow*. Survival curves were plotted with the Kaplan–Meier method. Overall survival was estimated after a log rank test. *p* < 0.05 was considered significant.

C‐reactive protein decreased significantly between Days 0 and 7 after CCP infusion (101 (CI 79–124) versus 37 (CI 25–48) mg/L, *p* < 0.0001). At Day 7, 48 patients (64%) had an improved condition. Among them, 16 (21%) were discharged from hospital. Among the 27 patients (36%) who experienced a worsening condition, seven (9%) were transferred to intensive care unit, and six (8%) died. At Day 28, the OS was 76% (95% CI = 67–86) (Figure 1A). The 28‐day OS was similar in the three groups of patients: 76% (95% CI = 65–88) for HM patients, 80% (95% CI = 59–100) for AID patients, and 72% (95% CI = 51–100) for SOT recipients (*p* = 0.9) (Figure 1B). The previous administration of anti‐CD20 monoclonal antibody (mAb) did not impact the 28‐day OS (*p* = 0.81) (Figure 1C). However 28‐days OS was higher in patients with WHO scale 5 compared to WHO scale 6 at the day of CCP infusion (88% [95% CI = 79–97] vs. 52% [95% CI = 35–77], *p* = 0.0009) (Figure 1D). No safety concern has been reported in the patients during the course of treatment.

## 4 Discussion

We report herein an observational cohort of immunosuppressed patients, mostly with HM (almost all with B‐lymphoid disease), infected by the Omicron subvariant BA.1 or BA.2, and treated with CCP. The 28‐day OS for the whole cohort was 76% without differences according to the type of underlying immunosuppression. In the recent observational study from the EPICOVIDEHA registry, mortality rate among hospitalized HM patients infected with Omicron was 16.5% and reached 23% 30‐day mortality in patients with chronic lymphoid leukemia (CLL) [11]. Of note in our cohort, 32% of patients needed high‐flow oxygen at the time of CCP infusion that could explain the lower response after CCP infusion compared to that reported in the EPICOVIDEHA registry. Surprisingly, there were no differences between patients previously treated or not with anti‐CD20 mAb. This is to note that almost all patients (89%) have anti‐S antibodies below 264 BAU/mL at the time of CCP infusion suggesting that not only antiCD20 mAb impair humoral response but a large panel of diseases or immunosuppressive drugs used to manage autoimmune disorders or limit graft rejection. Consistently, the beneficial effect of CCP has already been described in patients with primary antibody deficiency associating clinical improvement and decreased SARS‐CoV2 viral load [12]. In SOT recipients, few data are available, but two relevant series of 10 and 13 patients have reported the feasibility of CCP with a mortality rate of 10% and 23%, respectively, linked to COVID‐19 [13, 14].

To date, the time of CCP infusion remains debated. While B‐depleted patients on high‐flow oxygen and treated with CCP usually have a poor outcome [7], patients who present with a prolonged COVID‐19 could be treated before they need high‐flow oxygen [15]. Indeed, clinicians must distinguish patients with protracted disease as “smoldering COVID‐19” from patients presenting with an aggressive disease. Besides the time between CCP transfusion and symptoms onset is not so stereotypical as in immunocompetent patients [16], the disease course can therefore be informative and must be taken into account in the decision to use CCP.

The severity grade using the WHO scale was also associated with the outcome. Since the WHO scale may be influenced by other comorbidities, the global severity of COVID‐19, especially the level of oxygen need at the time of CCP infusion, may significantly affect the patients' outcome. Indeed, patients on high‐flow oxygen had a poor outcome despite CCP infusion with only 52% 28‐day OS.

Our study presented some limitations. First, the cohort is quite small, and the data collection is of retrospective nature. Also, we did not have a control group because patients were treated according to an early access program that makes difficult to obtain a comparable group and avoid any prescription bias. Indeed, the patients who were not eligible for CCP transfusion after CCP request were very different from those who were treated with CCP: They were not immunocompromised or had a differential diagnosis than COVID‐19. This precluded us to perform an exposed/non‐exposed analysis with a propensity score.

In a context of urgent need for therapeutic options when new SARS‐CoV‐2 variants emerge and escape current monoclonal antibodies, CCP remains a feasible and safe treatment option for immunosuppressed patients whatever the underlying disease. Some effort must be made to better anticipate the course of the disease course and guide the timing of CCP infusion. Randomized clinical trials are mandatory to better define the best setting in which to use CCP.

## Author Contributions

**Quentin Richier:** Conceptualization; Data curation; Formal analysis; Methodology; Writing – original draft. **Benjamin De Valence:** Conceptualization; Data curation; Writing – original draft. **Dorothée Chopin:** Data curation; Writing – review and editing. **Emmanuelle Gras:** Data curation; Supervision; Visualization; Writing – review and editing. **Laura I Levi:** Data curation; Visualization; Writing – review and editing. **Yasmine Abi Aad:** Writing – review and editing. **Jérôme Pacanowski:** Data curation; Writing – review and editing. **Jean‐Luc Meynard:** Data curation; Writing – review and editing. **Léo Plaçais:** Formal analysis. **Dorothée Fey:** Data curation. **Priscille Couture:** Data curation. **Guillaume Martin‐Blondel:** Data curation; Supervision; Writing – review and editing. **Vincent Pestre:** Data curation. **Juliette Woessner:** Data curation. **Sophie Ancellin:** Data curation. **Pierre Weyrich:** Data curation. **Benjamin Carpentier:** Data curation. **Salim Idri:** Conceptualization; Writing – review and editing. **Pierre Tiberghien:** Conceptualization; Writing – review and editing. **Laure Surgers:** Writing – review and editing. **Thomas Hueso:** Conceptualization; Formal analysis; Methodology; Supervision; Validation; Writing – original draft; Writing – review and editing. **Karine Lacombe:** Conceptualization; Data curation; Investigation; Supervision; Validation; Visualization; Writing – review and editing.

## Conflicts of Interest

QR, BDV, LP, YAA, LL and TH declare no conflicts of interest. KL has received funds from Gilead, MSD, Janssen, ViiV Healthcare, and Abbvie for expert boards and travel grants. LS has received a travel grant from Pfizer but with unrelated to COVID‐19.

### Peer Review

The peer review history for this article is available at https://www.webofscience.com/api/gateway/wos/peer‐review/10.1111/irv.13272.

## Data Availability

The data that support the findings of this study are available on request from the corresponding author. The data are not publicly available due to privacy or ethical restrictions.
